# Assessing the shooting distance of lead-free ammunition regardless of composition using Laser Induced Breakdown Spectroscopy

**DOI:** 10.1093/fsr/owad022

**Published:** 2023-08-03

**Authors:** Alicia Doña-Fernández, Jose Antonio Rodriguez-Pascual, Israel de Andres-Gimeno, Esperanza Gutiérrez-Redomero, Eduardo Valtuille-Fernández, Francisco Javier Gomez-Laina

**Affiliations:** Defence and Security System (Indra), Torrejón de Ardoz, Madrid, Spain; Ballistics Section of the Spanish Scientific Police Headquarters (National Police), Julián González Segador s/n, Madrid, Spain; Instituto Universitario de Investigación en Ciencias Policiales (IUICP), Universidad de Alcalá, Alcalá de Henares, Madrid, Spain; Chemical Laboratory of the Spanish Scientific Police Headquarters (National Police), Julián González Segador s/n, Madrid, Spain; Instituto Universitario de Investigación en Ciencias Policiales (IUICP), Universidad de Alcalá, Alcalá de Henares, Madrid, Spain; Departamento de Ciencias de la Vida, Universidad de Alcalá, Alcalá de Henares, Madrid, Spain; Center of Excellence in Security Systems (Indra), Moisés de Leon, Leon, Spain; Ballistics Section of the Spanish Scientific Police Headquarters (National Police), Julián González Segador s/n, Madrid, Spain; Instituto Universitario de Investigación en Ciencias Policiales (IUICP), Universidad de Alcalá, Alcalá de Henares, Madrid, Spain

**Keywords:** lead-free ammunition, LIBS, shooting distance determination, gunshot residue, crime scene, iForenLIBS system

## Abstract

At present, it is challenging to accurately determine firearm shooting distances in the case that lead-free ammunition is involved, largely because different manufacturers use different primer compositions. Laser-induced breakdown spectroscopy (LIBS) allows the simultaneous detection of multiple elements with high sensitivity and so may represent a solution to this problem. Previous studies have, in fact, demonstrated that LIBS can be used to determine shooting distances when working with gunshot residues from conventional ammunition based on scanning fabric surfaces. The present study confirms that the shooting distance can be ascertained using LIBS to detect copper originating from the ammunition casing and projectile but not the primer on fabric surfaces. This estimation can be performed regardless of the primer composition of lead-free ammunition.

**Key points:**

- Evaluation of gunshot residue from lead-free ammunition using scanning electron microscopy-energy dispersive X-ray analysis indicated that 40% of the particles contained copper.
- The iForenLIBS system allowed the detection of copper-containing particles on fabric surfaces after firing at different distances with high sensitivity.
- Laser-induced breakdown spectroscopy can determine the shooting distance of lead-free ammunition through copper detection even in ammunition that does not used this element in the primer.
- This technique can generate density maps allowing the evaluation of short, medium, and long-range shooting distances.

## Introduction

Lead is a toxic substance and so can pose a threat to both human health and the environment. The use of this metal is therefore highly regulated, and police forces are now required to employ ammunition in which both the projectile and primer do not contain lead. However, because conventional ammunition continues to be available, this creates a challenge for forensic laboratories with regard to the analysis of gunshot residues (GSR), particularly when the type of ammunition is unknown. This difficulty results in increased analysis time and laboratory costs.

Conventional ammunition is defined as the ammunition in which the primer comprises primarily lead styphnate, barium nitrate, and antimony sulphide. Once fired, those classified as characteristics of GSR particles with the elemental composition: antimony (Sb), lead (Pb), and barium (Ba) are formed. In addition, consistent particles with GSR (Pb–Ba–Ca–Si, Ba–Ca–Si, Sb–Ba, Pb–Ba, Sb–Pb, and/or Ba-Al) can be found [1]. By contrast, lead-free or nontoxic ammunition (NTA) can contain gadolinium (Gd), titanium (Ti), zinc (Zn), tin (Sn), silicon (Si), aluminium (Al), phosphorus (P), sulphur (S), chlorine (Cl), potassium (K), calcium (Ca), and other elements. The specific composition depends on the brand of ammunition because there is no consensus regarding the optimal formulation and so each manufacturer uses a different formulation. This situation has led researchers to perform studies intended to identify these new GSR particles and to provide new classifications. Characteristic GSR particles are considered as those that are composed of gadolinium, titanium, zinc or gallium, copper, and tin [1]. Romanò et al. [2] assessed both the chemical composition and morphology of GSR specimens obtained from NTA using scanning electron microscopy with energy dispersive X-ray analysis (SEM–EDX). This prior work determined that additional combinations of elements, such as Ti–Zn–K–Cu–Zn and Al–Si–K–S–Cu–Zn, could also be considered as GSR particles [2]. To facilitate the detection of heavy metal-free ammunition, brands such as Fiocchi have modified their formulation by introducing samarium oxide and titanium oxide to allow for easy identification, resulting in Sm–K–Si–Ti–Ca–Al-type particles. These GSR particles having highly specific compositions are easily detectable and classified using SEM–EDX [3]. The composition and morphology of GSR generated by the Sellier & Bellot munition used in the present study have been evaluated in previous studies [4]. Using cathodoluminescence, these particles were found to contain O, Si, K, Al, S, and Na, with high levels of Si and lower concentrations of K, Al, Ca, and S. In addition to these elements, Cu is often found as metallic droplets on the surfaces of GSR particles.

Chemographic colour tests are most commonly used to estimate the shooting distance associated with GSR. As an example, the sodium rhodizonate staining method is widely used in forensic laboratories to visualize the GSR patterns generated by conventional ammunition, based on the detection of lead and barium, and this technique has been continuously optimized [5, 6]. Glattstein et al. [7] have also described the use of the modified Greiss test, in which the GSR is transferred using an adhesive, to analyse nitrite ions and smokeless powder residues. In addition, the Scientific Working Group for Firearm and Toolmark Identification Guidelines for Gunshot Residue Distance Determinations provide Dithiooxamide and 2-Nitroso-1-Naphthol tests that are specific for the detection of copper residues associated with copper-jacketed bullets [8]. A preliminary study by Polovkova et al. [9] proposed the visualization of copper from SINTOX-marked ammunition based on the precipitation reaction of copper with rubeanic acid, while zinc and titanium from lead-free ammunition can be detected via a modified dithizone test [10]. Unfortunately, each of these colourimetric techniques has the disadvantage of requiring a subjective assessment of colour changes and so is dependent on the expertise and experience of the analyst and may also be affected by interfering substances such as blood.

Various studies have attempted to devise more objective methods of determining the shooting distance by using analytical techniques such as neutron activation analysis [11], atomic absorption spectroscopy [12], or inductively coupled plasma-mass spectrometry [13]. X-ray diffraction, combined with multivariate analysis [14], has provided good predictive models that are accurate to within ~3%–7% or 14% if data from the two firearms are combined. Elemental mapping based on milli-X-ray fluorescence analysis can also be employed to determine the shooting distance [15] by finding the distribution of iGSR particles.

At present, laser-induced breakdown spectroscopy (LIBS) is widely used in forensic analysis [16–20], especially in the analysis of GSR [21]. Studies have shown that this technique can be used to detect GSR particles on the hands of a person who has operated a firearm [22–24] and on various other surfaces [25]. LIBS can also determine shooting distances based on GSR from conventional ammunition [26, 27]. Fambro et al. [28] conducted preliminary studies, confirming that LIBS can serve as a rapid method for the preliminary screening of lead-free ammunition in conjunction with confirmatory testing by SEM–EDX. For the present study [29], a field and laboratory equipment based on LIBS technology (iForenLIBS system) has been employed. The Spanish Scientific Police Headquarters validated the GSR detection protocols of this system. iForenLIBS has been shown to be capable of detecting GSR particles based on the simultaneous analysis of Sb, Pb, and Ba even in the case that only a single particle having a diameter >1 µm is available. Other studies have also demonstrated the viability of LIBS as an approach to determining shooting distances [26, 27] and in the reconstruction of firearm-related incidents [25]. Such research has led to the incorporation of LIBS in their internal forensic protocols.

The Chemical Laboratory and Ballistics Department of Spanish Scientific Police continually assesses all commercially available ammunition. At present, they are evaluating the different GRS particles generated by new lead-free ammunition. Interestingly, studies conducted using SEM–EDX and the iForenLIBS system have confirmed high copper concentration in GSR samples obtained from such ammunition. In prior work, Merli et al. [30] evaluated the inorganic residues produced by three different copper-/zinc-jacketed bullets using inductively coupled plasma-optical emission spectrometry and XRF as a means of identifying specific ammunition.

The aim of the present study was to assess the possibility of using copper to determine firing distances in association with lead-free ammunition via analyses based on LIBS. Three cartridge cases having different compositions (manufactured by Sellier & Bellot, Ruag SWISS P SeCa, and Fiocchi Munizione) were previously analysed by SEM–EDX to identify the chemical elements present in GSR particles. In these trials, rounds were fired into white cotton fabric from distances varying between 8 and 200 cm, after which the residual copper on the fabric surfaces was analysed using the LIBS system to confirm automatic GSR detection and shooting distance estimation.

## Materials and methods

All sample preparations and analyses were carried out in the Chemical Laboratory and Ballistic Section of the Spanish Scientific Police Headquarters (National Police).

### Material

**Figure 1:**
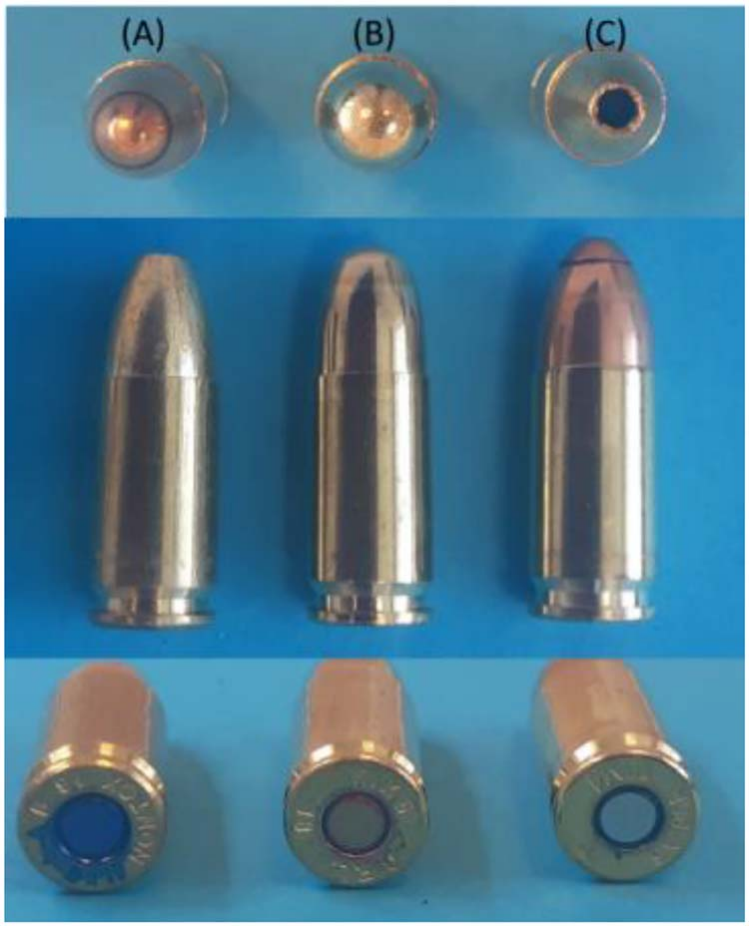
Images of the ammunition used (A) Sellier & Bellot, (B) Fiocchi, and (C) SeCa cartridges.

White cotton T-shirts and cardboard were employed as the target materials. Aluminium collection stubs, each with a diameter of 12.7 mm and carbon adhesive, were obtained from Agar Scientific (Essex, UK). All trials were performed using a shooting bench together with a laser distance meter. An H&K USP Compact semi-automatic pistol (Heckler and Koch GMBH, Oberndorf, Germany) was employed to fire the ammunition. Three types of ammunition (boxer system) were used. The first comprised Sellier & Bellot-20 9 × 19 mm NONTOX bullets (115 grs/7.5 g) as shown in Figure 1A. This was a homogenous service ammunition-monoblock projectile with a lead-free primer, CuZn10. The second type was GFL C1-18 9 × 19 mm NOTOX, full metal core 8G SOF, as shown in Figure 1B, with fully encapsulated bullets and a zero pollution heavy metal-free primer, CuZn30. The third type was RUAG SWISS P SeCa 9 × 19 mm (99 grs/6.4 g), as shown in Figure 1C. The latter ammunition was entirely lead-free, with full-jacketed bullets consisting of two tombac jackets slid into one another and combined with a lead-free ignition element, CuZn5.

These ammunition types were selected to ensure variations in the lead-free primer and also because all used different projectile types and jackets (Figure 1).

Disposable tips (iForenLIBS) and disposable plastic support for the automatic platform were used to prevent cross-contamination between samples.

### Test procedures and sample collection

The ammunition was fired at the white cotton T-shirts using the cardboard sheets as supports. In each case, a single shot was fired at each sample at a 90° angle of incidence using the H&K USP Compact pistol. This firearm was cleaned prior to the study to avoid any residual contamination from prior use with conventional ammunition (memory effect) and was also cleaned between each use of different lead-free ammunition.

**Figure 2:**
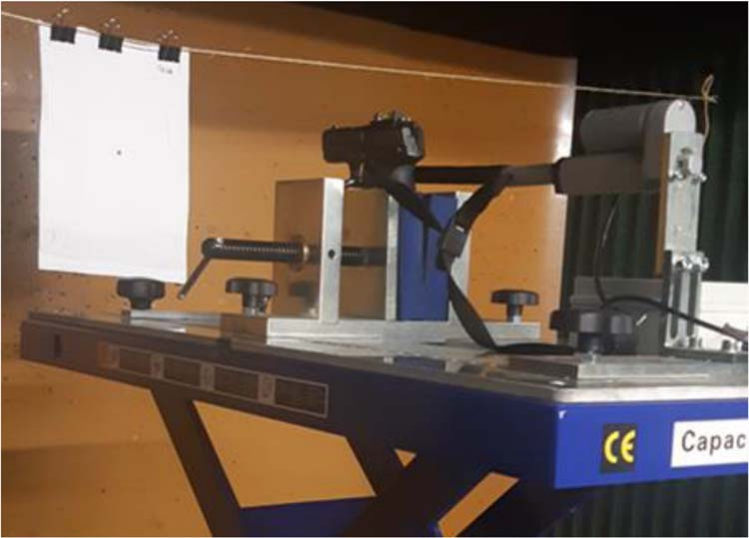
Image of the set-up carried out to obtain the shots at different distances.

Each firing was performed in a shooting gallery by specialists under controlled environmental conditions, and the facility was vacuum cleaned to avoid cross-contamination. To ensure consistency, a shooting bench with a laser distance meter was employed (Figure 2). The shots were fired in the order of longest to shortest distance. Shots were fired from distances of 8, 15, 20, 50, 75, 100, 140, and 200 cm with two trials at each distance, resulting in a total of 48 shots.

### Scanning electron microscopy with energy dispersive X-ray analysis

SEM–EDX analyses were used to estimate the number of copper-containing GSR particles generated by each trial with each type of ammunition. These analyses used a Zeiss EVO 10MA instrument (Jena, Germany) equipped with an Xmax50 Oxford detector, a tungsten filament, and special software intended for the analysis and classification of GSR.

### Sample collection

For each type of ammunition, a single shot was fired at a distance of 8 cm from a cotton T-shirt by using the protocol described in test procedures and sample collection. After firing, adhesive carbon was applied in a circular pattern with a radius of 5 cm around the entry hole to preserve the sample for further analysis. Subsequently, all stubs were covered with a 10-nm layer of carbon.

The SEM–EDX analyses were performed using an acceleration voltage of 20 kV, with a minimum particle size of 0.9 μm and processing time of 4. An Au–Rh–Co–C sample was used to calibrate the instrument. A minimum of two pixels were employed per particle with a magnification of 233×, and a circular geometry search was applied. The complete surface of each stub was automatically assessed using the particle analyser programme within the AZtec Oxford software (https://nano.oxinst.com/products/azteclive). This process incorporated a particle size limit to avoid acquiring an overly large volume of data so that the amount of copper examined in each sample was representative of the total copper in the sample.

### LIBS system

**Figure 3:**
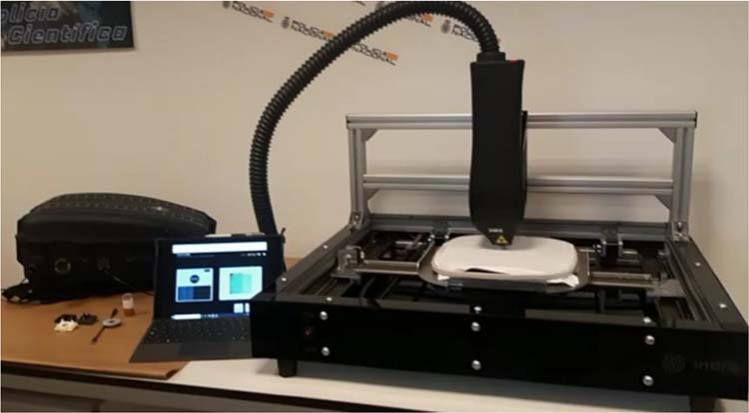
Image of the iForenLIBS system and the automatic platform during the scanning of a sample.

The Indra System iForenLIBS V.1 instrument was employed in this work. This equipment employs an Nd:YAG 1 064 nm laser providing an energy density in excess of 6 GW/cm^2^ that can be adjusted according to the sample type. The laser is focused on the sample surface through internal optics with a fixed spot size having a diameter of 500 μm. This device also incorporates a set of spectrometers with a total spectral range of 225–960 nm and an average resolution of 0.1 nm, enabling simultaneous analysis of all elements in the sample. The system offers various working modules, each with preconfigured analysis conditions for optimal signal-to-noise ratio and detection results. The iForensLIBS device can automatically detect 48 elements to provide qualitative and semi-quantitative results for sample compositions in a single analysis. This allows analysis of elements typically found in GSR from both conventional and lead-free ammunition, including Pb, Na, Sb, Ba, Sn, Ni, Al, Si, B, Cu, Ti, K, Gd, and Zn. This apparatus can be docked onto a platform to allow for automated scanning, and its open design allows for the testing of large samples (Figure 3).

### Analysis protocol

Each sample was securely placed on a disposable plastic support and was carefully transferred to the platform tray of the system. A camera located in the head of the system was used to centre the sample after which an automated scanning process was initiated. The head was designed to prevent direct contact with the samples, thus avoiding cross-contamination, or the transfer of particles between different areas of the same sample.

The Ballistic Module was selected, involving various preset conditions, to enable estimation of the shooting distance. The system scanned the surface of each specimen around the region at which the bullet penetrated to assess the entire region while moving around eight axes, resulting in a total of 2 917 shots. Once the scanning process was complete, the system generated a map of the relative concentrations (arbitrary unit). Artificial intelligence algorithms then provided an estimate of the shooting distance by comparing the data to the internal patterns in the system library. These internal patterns were pseudo-patterns integrated into the database rather than patterns previously acquired by the user. In addition to the results obtained in this study by comparing with these internal patterns, the system allows the use of the same algorithm with patterns acquired under specific conditions (ammunition, firearm type, etc.) that have been previously analysed. The user can select these patterns from the library.

**Table 1:** Statistics related to the evaluation of shooting distance (*N* = 211).

	Short distance (%)	Medium distance (%)	Large distance (%)
Sensitivity	91	94	98
False negative rate	9	6	2
Precision	98	95	93
Specificity	99.4	98	97
Accuracy	98	95	97
Negative predictive value	98	95	99
False positive rate	0.6	2	3

**Figure 4:**
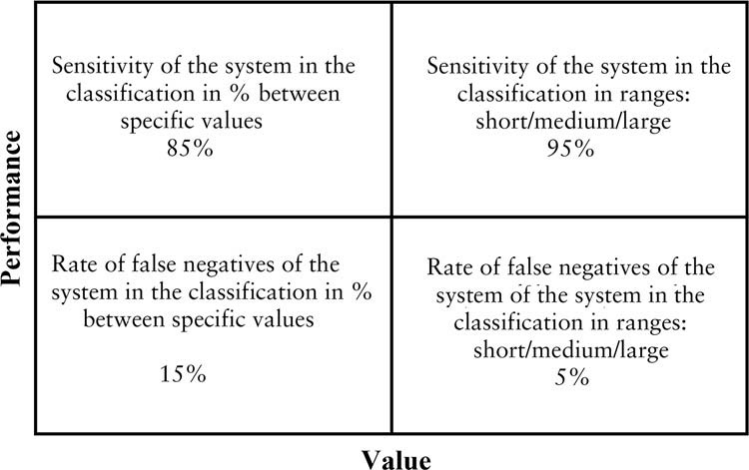
Performance values regarding the determination of shooting distance (*N* = 211).

**Figure 5:**
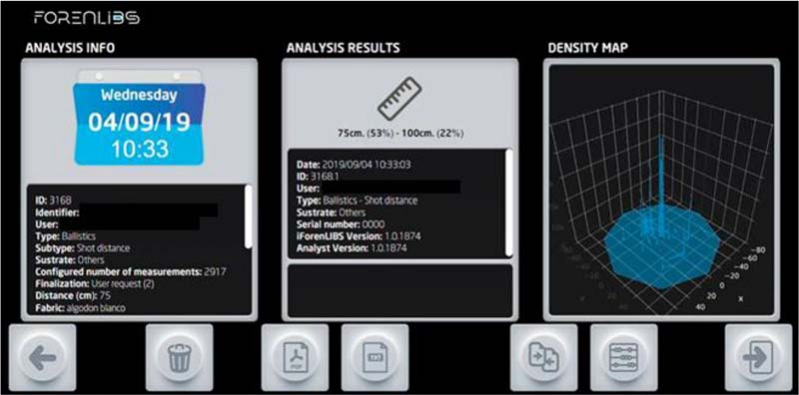
Image of the software interface with a display of the results of the shooting distance determination and the density map.

Table 1 and Figure 4 shows the statistical results of the capacity to estimate the shooting distance, as obtained and presented in previous studies [31].

Once the analysis was complete, the results were displayed on the device user interface (Figure 5). This display comprised a density map of the element being assessed (lead, boron, zinc, or copper), which allowed a visualization of the spatial arrangement of the GSR particles and their relative concentrations. This visual map permits identification of the possible entrance and exit holes through the difference in concentration as well as any anomalies in the GSR distribution, angulation, and other parameters. The results of this analysis could also be used to generate an estimate of the shooting distance by comparison with internal patterns and a “percentage similarity” after selection of a given element. As an initial approximation, the software indicated whether the apparent shooting distance could be categorized as short (defined as 8–25 cm), medium (25–100 cm), or long range (100–200 cm). The programme also indicated the percentage similarity within ranges defined as short (8–15–25 cm), medium (25–50–75–100 cm), or long (100–140–200 cm). The system database also guaranteed that the results obtained during each analysis complied with the chain of custody requirements.

## Results and discussion

### SEM–EDX analysis

**Figure 6:**
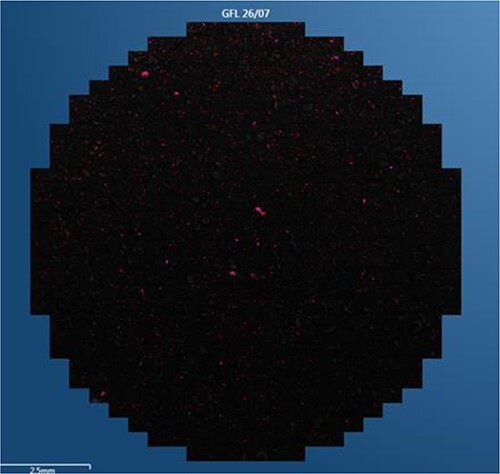
Distribution of copper (red spots) over a sample surface.

**Figure 7:**
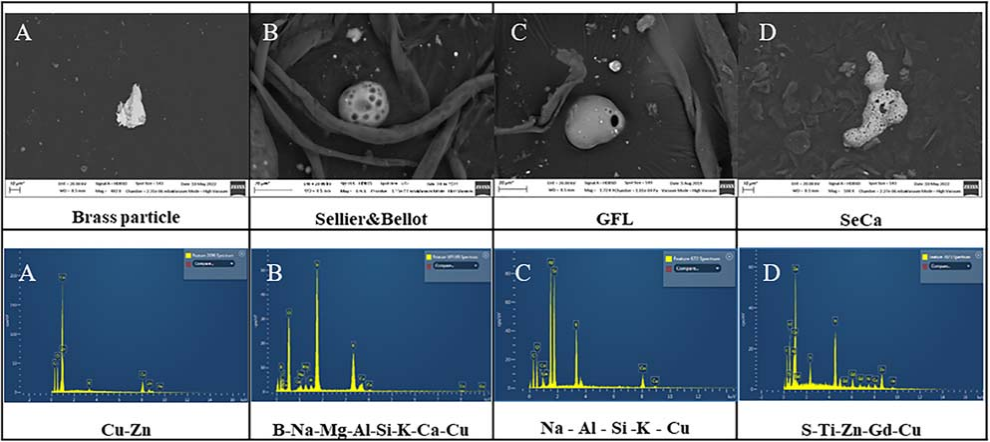
SEM images acquired at 482× magnification and an accelerating voltage of 20 kV with a working distance of 8.5 mm and EDX spectra of (A) brass, (B) Sellier & Bellot, (C) GFL, and (D) SeCa GSR particles.

The total quantities of particles detected in each trial using the SEM–EDX automatic search function and the total quantities of particles that contained copper are summarized in Table 2. Figure 6 presents an image showing the distribution of GSR particles containing copper produced by the Fiocchi ammunition. Here, the copper-containing particles appear red. The copper found in the various specimens was evidently in different forms, either as isolated particles of elemental copper or alloyed with other elements. The most common alloy produced by all ammunition types evaluated in this work was brass (CuZn) (Figure 7A). In the case of the S&B-20 NONTOX, the elements most commonly alloyed with copper were B, Na, Mg, Al, Si, K, Ca, Fe, and Cu. The copper could be combined with one or several or all of these elements (Figure 7B). The GFL-18 NOTOX generated particles in which copper was combined with one or more of Na, Al, Si, K, Ca, Fe, and Cu (Figure 7C). Finally, the trials using the RUAG SeCa provided particles comprising alloys of copper with Ti, Zn, Cu, Ni, S, K, Ni, and Gd (Figure 7D).

These results indicate that, during deflagration, the direct contact of the primer with the interior of the cartridge case (such as the flash hole), the primer cap (anvil), and the projectile produced GSR particles containing copper.

### LIBS analysis

As noted, not all lead-free ammunition uses the same chemical composition, which creates difficulties in determining the optimal method for GSR analysis and may necessitate successive colourimetric tests. Additionally, considering the memory of the weapon, simultaneous study of multiple elements might lead to an incorrect estimation due to contradictory images. The present work therefore simplifies the analytical process to reduce response time as well as cost by evaluating a single element (copper) independent of the chemical composition of the primer.

Previous SEM–EDX studies of particles have shown that copper is found in ~40% of the GSR particles generated by a cartridge. Thus, the present work examined the use of LIBS to ascertain the surface distribution of copper as a means of estimating shooting distance.

**Figure 8:**
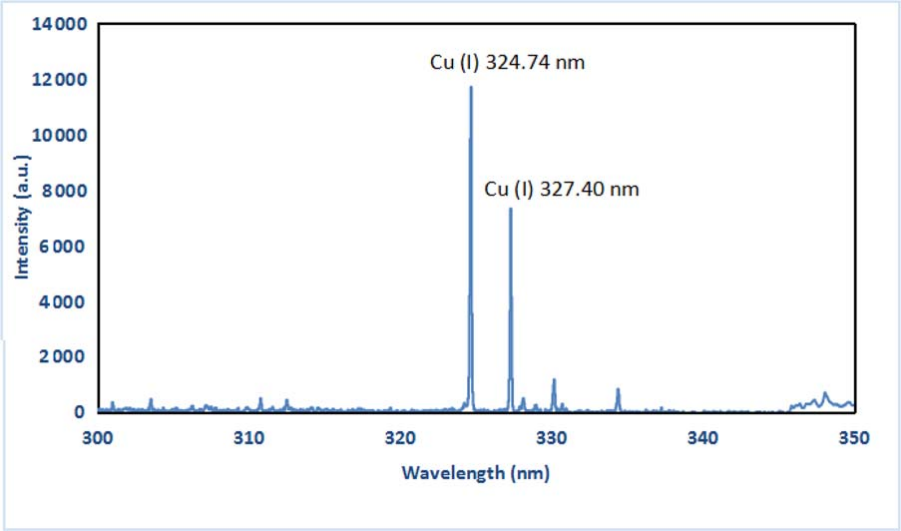
LIBS spectrum acquired from a cartridge case showing the atomic emission lines of copper.

The system performs a double check for copper identification using the atomic emission lines 324.75 and 327.40 nm. Figure 8 presents a typical spectrum obtained from a cartridge case analysis, which contains these emission lines. The relative concentration of copper was calculated based on the most intense peak at 324.75 nm.

**Table 2:** Particle quantities determined by SEM–EDX.

Ammunition	Total particles (*n*)	Copper *n* (%)
S&B-20 NONTOX	4 863	2 054 (42.24)
GFL-18 NOTOX	5 482	1 949 (35.55)
RUAG SeCa	4 174	1 597 (38.26)

**Figure 9:**
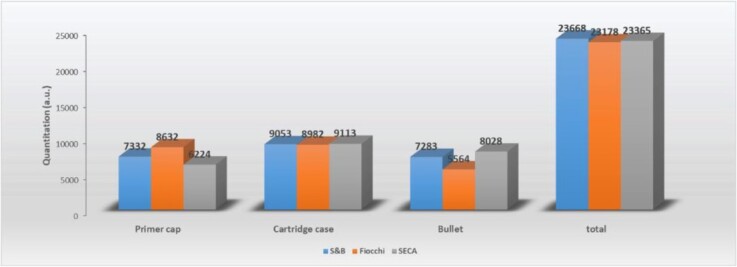
Copper quantification data for the various ammunition samples.

**Figure 10:**
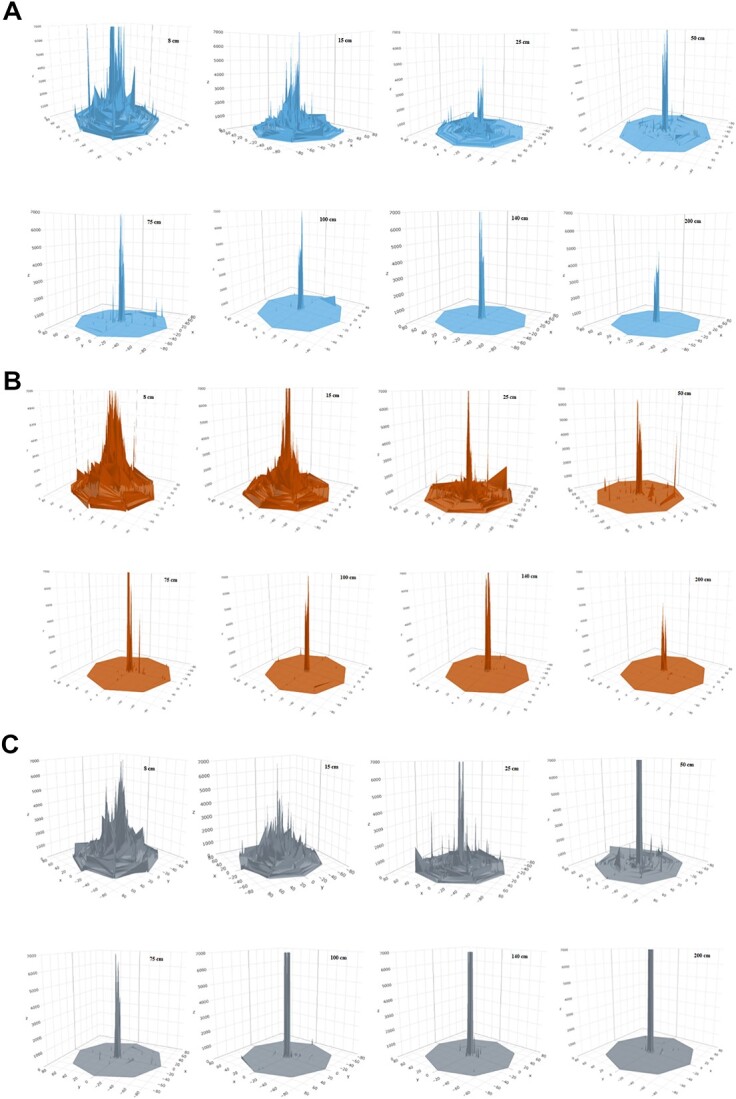
Copper density maps generated by the (A) Sellier & Bellot , (B) Fiocchi, and (C) SeCa ammunition, showing distributions and concentrations on the sample fabric at each of the distances.

**Table 3:** Ammunition samples analysed and results from the automated determination of shooting distance.

Sample	Ammunition	Real distance (cm)	iForenLIBS response (S/M/L)	iForenLIBS response (%)
1	S&B-20 NONTOX	8	Short distance	80% 8 cm–19% 15 cm
2	S&B-20 NONTOX	8	Short distance	32% 8 cm–45% 15 cm
3	S&B-20 NONTOX	15	Short distance	13% 8 cm–86% 15 cm
4	S&B-20 NONTOX	15	Short distance	25% 8 cm–70% 15 cm
5	S&B-20 NONTOX	25	Medium distance	92% 25 cm–3% 50 cm
6	S&B-20 NONTOX	25	Short distance	8% 15 cm–85% 25 cm
7	S&B-20 NONTOX	50	Medium distance	29% 50 cm–35% 75 cm
8	S&B-20 NONTOX	50	Medium distance	37% 50 cm–22% 75 cm
9	S&B-20 NONTOX	75	Medium distance	49% 75 cm–27% 100 cm
10	S&B-20 NONTOX	75	Medium distance	34% 75 cm–39% 100 cm
11	S&B-20 NONTOX	100	Medium distance	34% 75 cm–56% 100 cm
12	S&B-20 NONTOX	100	Medium distance	31% 75 cm–59% 100 cm
13	S&B-20 NONTOX	140	Large distance	32% 140 cm–45% 200 cm
14	S&B-20 NONTOX	140	Large distance	66% 140 cm–17% 200 cm
15	S&B-20 NONTOX	200	Large distance	28% 140 cm–52% 200 cm
16	S&B-20 NONTOX	200	Large distance	30% 140 cm–49% 200 cm
17	GFL-18 NOTOX	8	Short distance	78% 8 cm–20% 15 cm
18	GFL-18 NOTOX	8	Short distance	57% 8 cm–24% 15 cm
19	GFL-18 NOTOX	15	Short distance	11% 8 cm–89% 15 cm
20	GFL-18 NOTOX	15	Short distance	13% 8 cm–87% 15 cm
21	GFL-18 NOTOX	25	Short distance	15% 15 cm–81% 25 cm
22	GFL-18 NOTOX	25	Short distance	50% 15 cm–35% 25 cm
23	GFL-18 NOTOX	50	Medium distance	42% 50 cm–23% 75 cm
24	GFL-18 NOTOX	50	Medium distance	36% 50 cm–25% 75 cm
25	GFL-18 NOTOX	75	Medium distance	47% 75 cm–33% 100 cm
26	GFL-18 NOTOX	75	Medium distance	36% 75 cm–46% 100 cm
27	GFL-18 NOTOX	100	Medium distance	19% 75 cm–70% 100 cm
28	GFL-18 NOTOX	100	Medium distance	44% 75 cm–45% 100 cm
29	GFL-18 NOTOX	140	Large distance	54% 140 cm–25% 200 cm
30	GFL-18 NOTOX	140	Large distance	45% 140 cm–28% 200 cm
31	GFL-18 NOTOX	200	Large distance	36% 140 cm–51% 200 cm
32	GFL-18 NOTOX	200	Large distance	43% 140 cm–42% 200 cm
33	RUAG SeCa	8	Short distance	90% 8 cm–7% 15 cm
34	RUAG SeCa	8	Short distance	78% 8 cm–14% 15 cm
35	RUAG SeCa	15	Short distance	30% 8 cm–51% 15 cm
36	RUAG SeCa	15	Short distance	22% 8 cm–76% 15 cm
37	RUAG SeCa	25	Short distance	55% 15 cm–28% 25 cm
38	RUAG SeCa	25	Short distance	50% 15 cm–36% 25 cm
39	RUAG SeCa	50	Medium distance	51% 50 cm–16% 75 cm
40	RUAG SeCa	50	Medium distance	38% 50 cm–22% 75 cm
41	RUAG SeCa	75	Medium distance	40% 75 cm–37% 100 cm
42	RUAG SeCa	75	Medium distance	39% 75 cm–41% 100 cm
43	RUAG SeCa	100	Medium distance	40% 75 cm–42% 100 cm
44	RUAG SeCa	100	Medium distance	24% 75 cm–59% 100 cm
45	RUAG SeCa	140	Large distance	65% 140 cm–25% 200 cm
46	RUAG SeCa	140	Large distance	43% 140 cm–30% 200 cm
47	RUAG SeCa	200	Large distance	27% 140 cm–49% 200 cm
48	RUAG SeCa	200	Large distance	36% 140 cm–44% 200 cm

#### Analysis of Cu concentrations in ammunition

The compositions of the cartridge cases, primer caps, and projectiles of the three types of ammunition used in this work were evaluated. In this process, these cartridge parts were separated and cleaned using 96% ethanol after which each component was analysed using the manual ballistics module, which involved firing 50 shots. The analysis was conducted directly on the samples and in-depth without the need for any prior preparation. The anvil of each primer cap, which was in direct contact with the primer during ignition/deflagration, was also analysed. The graph in Figure 9 summarizes the average copper concentrations (in arbitrary units) for each component and each type of ammunition. The SeCa cartridges were found to have higher copper concentrations in the projectile but lower concentrations in the primer caps. By contrast, the Fiocchi ammunition had lower copper concentrations in the projectile but higher concentrations in the primer caps. All three types had the same copper concentrations in their cases. It is also evident from these data that the total copper balance was similar for all three types of ammunition.

#### Assessment of the surface concentrations and distributions of copper

Sixteen samples obtained by firing at different distances were analysed for each type of ammunition using the protocol described in analysis protocol. The shooting distance determination module of the equipment automatically analysed each sample, and the total analysis and processing time for each sample was 140 min. The density maps generated by the system indicate that copper particles were detected and had a characteristic distribution for each distance and for each type of ammunition. Representative data for the Sellier & Bellot, Fiocchi, and SeCa trials are provided in Figures 10. The system’s software was able to differentiate and estimate the shooting distance based on these distributions.

In these density maps, the *z*-axis indicates the relative copper concentration. A similarity in the spatial distributions of the particles can be observed between the three types of ammunition samples assessed for each distance. This is attributed to the lack of significant variations in the copper content between the different types of ammunition used (Figure 9). Due to the sensitivity of the system, even at large distances, a slight dispersion of the copper can be seen on the surfaces. A variation in the concentration of copper is observed in the hole, which is the area in direct contact with the projectile (cleaning area). This is due to the different compositions of the brass used in the bullets of each ammunition.

Table 3 shows the automatic results obtained from the shooting distance estimation. These data include initial approximations indicating whether the shot was fired at a short, medium, or long range, and the results show that all samples were classified correctly by the automated system. It should be noted that Sample 5, representing S&B ammunition fired from a distance of 25 cm, was determined by the system to be a medium-distance sample. However, in this case, the evaluation is considered to be correct because this was the cut-off value and so the result could fall into two different categories. In addition, a high percentage value was obtained.

The software also generated a second value in the form of a percentage (Table 3). This second level of classification only compares the distances within a given range by assigning a percentage value to each. A result is considered to be correct when the real distance falls between the distances, with the maximum percentage values indicated by the system for short distances, and the total (100%) is distributed among the three values included in the range (8, 15, and 25 cm). For medium distances, it is distributed among the four values included in the range (25, 50, 75, and 100 cm). For long distances, it is distributed among the three values included in the range (100, 140, and 200 cm). The percentage values presented in the Table 3 correspond to the two maximum values obtained from comparisons with the internal patterns of the distances included in each range.

The samples fired at short distances were all classified within the appropriate ranges. Within this group, the SeCa ammunition results tended to have shorter values. This effect can possibly be attributed to the higher concentration of copper in the entry hole resulting from the specific composition of the projectile, as shown in the graph in Figure 9.

At medium distances (Table 3), the data fall within the expected ranges, although the percentage values are slightly lower. In some cases, the values are distributed between the closest distances, as seen in Sample 26, which was shot at 75 cm, where the software classified this sample as being 36% at 75 cm and as being 46% at 100 cm. The system also exhibited the capacity to detect the copper deposited after long-distance firing, which allows for more precise classification. The percentage values obtained with all three types of ammunition fall within the established ranges.

The system also provided the option to choose other chemical elements to assess the shooting distance. In the case of lead-free ammunition, the distributions of these elements unique to each type of ammunition—boron (Sellier & Bellot), zinc (SeCa), and aluminium (Fiocchi)—could potentially be determined in addition to the copper density maps (Figure 10). These supplementary data are highly beneficial as they assist in identifying the type of ammunition used in each scenario and can be used to verify the shooting distance obtained by analysing copper.

## Conclusions

SEM–EDX analyses established that ~40% of the GSR particles generated by firing lead-free ammunition contained copper. Assessments of primer caps, cartridge cases and projectiles using LIBS showed similar relative copper concentration values for all three ammunition types used in the study. The distributions of copper-containing GSR particles on fabric surfaces were also observed, and the high sensitivity of the LIBS system allowed the detection of these particles even with a shooting distance of 2 m.

The characteristic density maps obtained from trials with all three munitions at all distances enabled the determination of the shooting distances. The images obtained from the different ammunition types were comparable, making it possible to determine the distance without prior knowledge of the ammunition used. The LIBS system demonstrated its capacity to ascertain the shooting distance based on the analysis of copper, thus providing an objective approach to GSR assessment.

The study demonstrated the potential of using copper to determine the shooting distance using the LIBS technique for lead-free ammunition, which does not contain copper in its primer. Although the present study focused on copper because this element was common among the three munitions, LIBS can simultaneously analyse numerous other elements contained in various primers. Such assessments could provide verification of the primer compositions and enable visualization of the spatial distribution of these elements, which could be crucial in the examination of crime scenes involving multiple firearms.

To further corroborate the results obtained, it will be necessary to evaluate a larger range of lead-free ammunition types. However, the promising results obtained in this study demonstrate the potential of using copper to determine shooting distances, thus providing a valuable tool for forensic investigations.
